# Recognition of Upper Limb Action Intention Based on IMU

**DOI:** 10.3390/s22051954

**Published:** 2022-03-02

**Authors:** Jian-Wei Cui, Zhi-Gang Li, Han Du, Bing-Yan Yan, Pu-Dong Lu

**Affiliations:** School of Instrument Science and Engineering, Southeast University, Nanjing 210096, China; 220193290@seu.edu.cn (Z.-G.L.); 220203465@seu.edu.cn (H.D.); 220203464@seu.edu.cn (B.-Y.Y.); 220193325@seu.edu.cn (P.-D.L.)

**Keywords:** action intention recognition of upper limb, prosthetic hand control, inertial sensor, dividing motion unit, 10-fold cross-validation

## Abstract

Using motion information of the upper limb to control the prosthetic hand has become a hotspot of current research. The operation of the prosthetic hand must also be coordinated with the user’s intention. Therefore, identifying action intention of the upper limb based on motion information of the upper limb is key to controlling the prosthetic hand. Since a wearable inertial sensor bears the advantages of small size, low cost, and little external environment interference, we employ an inertial sensor to collect angle and angular velocity data during movement of the upper limb. Aiming at the action classification for putting on socks, putting on shoes and tying shoelaces, this paper proposes a recognition model based on the Dynamic Time Warping (DTW) algorithm of the motion unit. Based on whether the upper limb is moving, the complete motion data are divided into several motion units. Considering the delay associated with controlling the prosthetic hand, this paper only performs feature extraction on the first motion unit and the second motion unit, and recognizes action on different classifiers. The experimental results reveal that the DTW algorithm based on motion unit bears a higher recognition rate and lower running time. The recognition rate reaches as high as 99.46%, and the average running time measures 8.027 ms. In order to enable the prosthetic hand to understand the grasping intention of the upper limb, this paper proposes a Generalized Regression Neural Network (GRNN) model based on 10-fold cross-validation. The motion state of the upper limb is subdivided, and the static state is used as the sign of controlling the prosthetic hand. This paper applies a 10-fold cross-validation method to train the neural network model to find the optimal smoothing parameter. In addition, the recognition performance of different neural networks is compared. The experimental results show that the GRNN model based on 10-fold cross-validation exhibits a high accuracy rate, capable of reaching 98.28%. Finally, the two algorithms proposed in this paper are implemented in an experiment of using the prosthetic hand to reproduce an action, and the feasibility and practicability of the algorithm are verified by experiment.

## 1. Introduction

The human hand is one of the most important organs of the human body. It can perform tasks which include grasping, kneading and other actions according to human intentions. Unfortunately, some unexpected events in life, such as traffic accidents, factory production, and illness, can cause hand deformities. In more severe cases, this can lead to upper limb amputation and loss of the ability to perform daily activities, such as putting on shoes, dressing, and eating. A lack of hand functionality presents significant challenges to the daily lives of handicapped individuals. An artificial manipulator represents a kind of artificial hand with some appearance features and functions of the human hand, which can simulate the action of the human hand, and has become an effective extension of human limb function. Importantly, a significant application field of artificial manipulators is the assistance of the handicapped [1,2]. Handicapped individuals generally have sound intelligence, vision, hearing, etc. Therefore, the key to using a handicapped manipulator is to coordinate with the user’s action intention.

Collecting and analyzing motion information of the upper limb is the first step in identifying motion intention. Information sources containing motion intention information of the human body include bioelectrical signals, kinetic signals, and voice signals, wherein the bioelectrical signals mainly include electromyographic (EMG) signals, electroencephalographic (EEG) signals, and neural signals [3]. Myoelectric prosthesis is one of the most widely used prostheses at present. It mainly controls the prosthesis by acquiring EMG signals of the upper limb. The EMG signal bears a weak current (uV to mV level) generated during depolarization of the sarcolemma, and generation of the weak current triggers muscle contraction [4]. Therefore, EMG signals can be measured by placing conductors on the surface of the skin or inside the muscles. Ahmed et al. [5] captured EMG signals from patients undergoing transhumeral amputation and used feature extraction to design a control method for EMG prostheses. Experiments have shown that this method is suitable for various patients. Mion et al. developed a wearable surface EMG biosensing system that can be used for real-time gesture classification. For 21 types of teacher recognition, the accuracy rate reached 92.87% [6].

Conscious human activity causes the nerve cells of the brain to generate rhythmic electrical signals. Electrodes placed on regions of the scalp can be used to detect EEG signals that reflect human consciousness activities, thereby controlling the movements of the prosthetic limbs. Yuriy et al. [7] developed a non-invasive brain–computer interface system based on EEG. The system detects users’ mental intentions from EEG data with an average accuracy rate of 80%. Koksal et al. [8] designed a new mathematical model and developed a voice control system, which was applied to the control of a four-joint manipulator. The experiments reveal that voice commands can be effectively employed to control the prosthetic manipulator. However, the use of voice control alone cannot meet the intelligent requirements of the handicapped individual to control the prosthesis with the mind. Therefore, EMG signals and voice signals are usually combined to meet the requirements of handicapped individuals for intelligent prosthetic control [9].

Although a myoelectric prosthesis possesses a wide range of applications, myoelectric control also bears certain limitations. The higher the degree of upper extremity amputation, the less limb muscle remains, resulting in fewer sources of EMG signals. Moreover, EMG signals are easily interfered with by skin sweat, epidermal hair, muscle impedance and external electromagnetic interference, which exhibit a certain impact on EMG control [10,11,12]. Inertial sensors have the advantages of strong wearability, simple equipment use, and little external environment interference, and have been successfully used in behavior analysis [13], medical diagnosis of patients [14], rehabilitation therapy [15] and other fields. Of course, in the application of controlling prosthetics, inertial sensors also bear many successful cases. Agamemnon et al. [16] designed an end-to-end control strategy using EMG electrodes and an inertial measurement unit, and implemented a real-time pick and place experiment using a commercial multi-joint prosthetic hand. Krausz et al. [17] studied visual, EMG, inertial measurement unit, and other data in powered lower-limb prosthetic movements and analyzed intra- and inter-subject and activity variability for each sensor modality. The results may prove useful for future applications in forward predictors for powered lower limb prostheses and exoskeletons. Therefore, based on the above literature research, considering the prosthesis needs to meet the requirements of simple operation, convenient installation and use, etc., this study only uses inertial sensors to collect motion information of the upper limb and analyze the user’s action intention to control the manipulator.

Accurately identifying the user’s action intent presents another key issue. Action intent recognition is mainly divided into two steps, feature extraction and machine learning. The extracted features mainly include time domain [18], frequency domain [19], and time–frequency characteristics [20]. In the process of recognizing different actions, the classification effect of a single feature may be different. Another factor affecting action recognition is the choice of machine learning algorithm. Currently, commonly employed algorithms for recognizing action intent include support vector machines (SVM), decision trees, and neural networks. Tianyang Zhong et al. [21] used the time–frequency information fusion method to obtain the time–frequency features of surface EMG signals, and introduced a deep belief network to identify upper limb rehabilitation actions. Naiqiao Ning and Yong Tang [22] extracted surface EMG signals in time and frequency domains, and employed an optimized support vector machine to identify upper limb movements. Hussain et al. implemented inertial sensors to record the motion trajectory of the measured object, and used the long short-term memory to recognize physical activities and related action units [23]. Sun et al. applied inertial sensors to obtain attitude data of prosthetic knee joints, and realized adaptive speed control of prosthetic knee joints by using BP neural network [24].

The main purpose of the research is to identify the action intention of the upper limb by using the upper limb motion information collected by inertial sensors, and to design a control method of the manipulator, so that the manipulator can reproduce the foot movements of daily life. Therefore, this paper acquires healthy participants performing foot movements as the research object, and selects the movements of putting on socks, putting on shoes, and tying shoelaces. The motion information of the upper limbs is analyzed by using the time domain features, and then the action types of the upper limbs are identified by a dynamic time warping (DTW) algorithm based on the motion unit. Finally, a generalized regression neural network (GRNN) algorithm based on 10-fold cross-validation is employed to identify the grasping intention of the upper limb to accurately control the opening and closing of the manipulator.

The main contributions are as follows:

(1) According to the time sequence of the complete action, and whether the upper limb is moving, the continuous action of the upper limb is divided into several motion units. Based on the motion unit, the DTW algorithm is used to classify the actions of putting on socks, putting on shoes, and tying shoelaces, which will effectively reduce the running time of the algorithm and improve the accuracy of action classification.
(2) Divide the experimental data into a training set and a test set. According to the data of the training set, implement the 10-fold cross-validation method to train the smoothing parameters of the GRNN to identify the optimal parameters. Compared with the traditional GRNN, radial basis function neural network (RBFNN) and other network models, the optimized GRNN algorithm exhibits higher accuracy.

The structure of the paper is as follows: Section 2 introduces the collection and analysis methods of upper limb motion data in detail, including data preprocessing, feature extraction, and data window segmentation. Section 3 introduces the DTW algorithm based on motion unit, and the GRNN algorithm based on 10-fold cross-validation. Section 4 provides results and analysis to validate the performance of the method. Finally, conclusions are provided in Section 5.

## 2. Data Acquisition and Analysis

### 2.1. Data Platform and Acquisition

**Figure 1:**
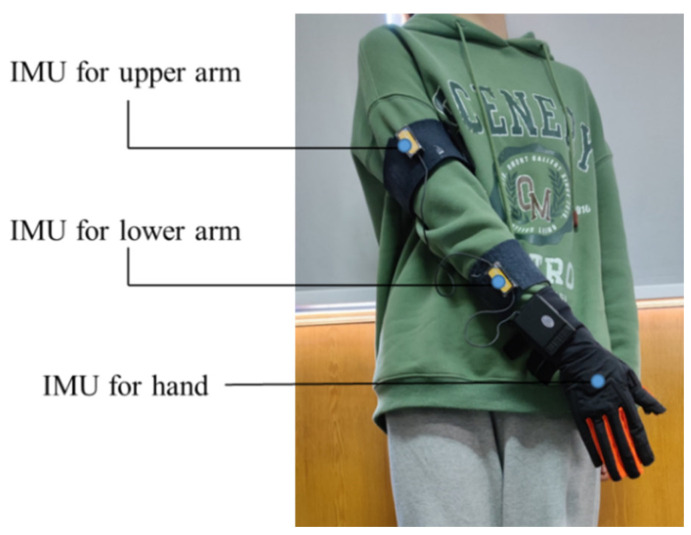
Sensor installation location.

In the process of collecting upper limb data, we used a data glove produced by Beijing Xintian Vision Technology Co., Ltd., Beijing, China. The data glove consists of three sets of inertial measurement units, including three-axis accelerometers and three-axis gyroscopes. The sensor collects information every 20 milliseconds, the frequency is 50 Hz, and the accuracy of the sensor is 0.2 degrees. Concerning practical use, three sets of inertial measurement units are installed on the upper arm, lower arm and hand, respectively. Figure 1 shows the installation position of the sensor. The data glove is used to collect the Euler angles and angular velocities of the three parts during the movement of the upper body. The pose data are transmitted to the computer through a serial port, and all the processes of data analysis are completed by the computer. Before using the IMU sensor, we need to calibrate the sensor using the calibration software and instructions provided by the manufacturer.

Sixty volunteers participated in this experiment, including 30 males and 30 females, ranging in age from 18 to 65 years old. The experiment of putting on socks, putting on shoes and tying shoelaces were performed by all volunteers. Each volunteer wore data gloves as required, and the experiment was repeated 50 times. A total of 3000 datasets were collected for each action type, resulting in an overall total of 9000 datasets. All volunteers underwent proficiency training before conducting the experiment.

### 2.2. Data Preprocessing

**Figure 2:**
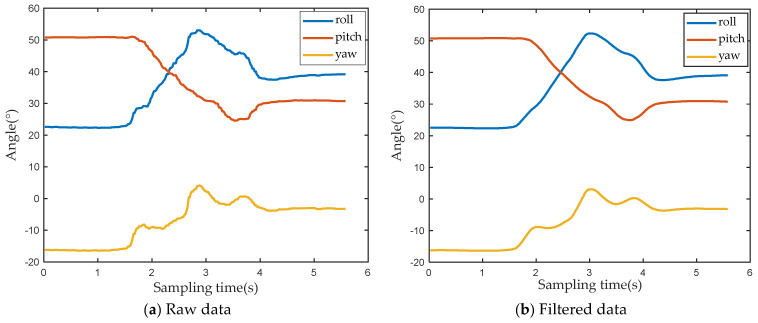
Data before and after filtering.

During use of the sensor, factors such as the external magnetic field environment, shaking of the arm, and long-term drift of the sensor may cause the collected data to contain noise. Figure 2a shows the raw angle data of the sensor. The blue curve represents the roll angle of the upper arm, the red curve represents the pitch angle of the upper arm, and the yellow curve represents the yaw angle of the upper arm. As can be seen from the figure, the original data contains some glitches and there is no impulsive interference. Therefore, this paper selects the moving average filtering method to filter the original data. Figure 2b displays the filtered data. Compared with the original data, in the entire sampling interval, the angle value curves of the three axes become smoother, and there are almost no burrs, indicating that the effect of noise is effectively reduced after filtering.

### 2.3. Feature Extraction

**Figure 3:**
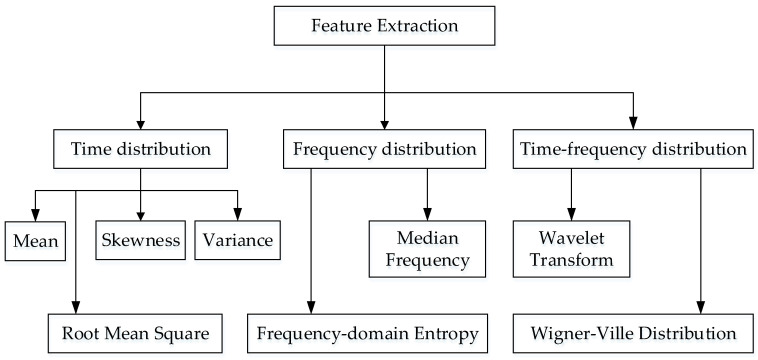
Processing method of inertial data.

When analyzing data collected by inertial sensors, features that are often extracted fall into three categories: time-domain features, frequency-domain features, and time–frequency features. Through feature extraction, it is easier to characterize the motion law of the upper limb, which helps to improve the accuracy of intent recognition. Ge Gao [25] compares the effects of time-domain features and frequency-domain features in action recognition, respectively. In dynamic actions, the frequency domain features employed bear a higher recognition rate; in static and excessive actions, the temporal features used have a higher recognition rate. Figure 3 presents the processing method for inertial data.

Considering the speed and real-time nature of data processing, this paper employs time domain features to analyze motion data of the upper limb, including the comprehensive value E, variance, difference, maximum and minimum values of the angular velocity of each part of the upper limb. Since the data collected by the inertial sensor represent the angular velocity of the x-axis, the y-axis, and the z-axis, during data analysis, the comprehensive value of the angular velocity of each part of the upper limb is obtained, defined as *E*. In the subsequent feature extraction process, the analysis is performed around the comprehensive value of the angular velocity of each part of the upper limb. Equation (1) defines the comprehensive value *E* of each part of the upper limb.

(1) $$E_{i}=\sqrt{{\left(\omega_{x}\right)}_{i}^{2}+{\left(\omega_{y}\right)}_{i}^{2}+{\left(\omega_{z}\right)}_{i}^{2}},i=1,2,3\ldots N$$

where ${\left(\omega_{x}\right)}_{i}$, ${\left(\omega_{y}\right)}_{i}$, ${\left(\omega_{z}\right)}_{i}$, respectively, represent the angular velocity of the x-axis, the angular velocity of the y-axis, and the angular velocity of the z-axis of the *i*-th sampling point of a certain part of the upper limb, *N* indicates the length of the sliding window.

Variance (VAR) can be used to measure the dispersion of a set of data. When analyzing the comprehensive value of the angular velocity of the upper limbs, the magnitude of the VAR can represent the degree of fluctuation of a set of data. The larger the VAR, the more violent the fluctuation, indicating that the range of motion of the upper limb is larger at this time; on the contrary, the smaller the VAR, the more gentle the fluctuation, indicating that the range of motion of the upper limb is smaller at this time. The equation for VAR is shown in Equation (2).

(2) $$\mathrm{VAR}=\frac{\sum_{i=1}^{N}{\left(E_{i}-\bar{E}\right)}^{2}}{N-1}$$

where $E_{i}$ represents the i-th comprehensive value of angular velocity in a set of data of a certain part of the upper limb, and $\bar{E}$ represents the average value of this set of data.

Differentiate the angular velocity to obtain the angular acceleration. The magnitude of the angular acceleration can characterize the speed of change in the speed of the object’s movement. Therefore, in a set of data, the difference between two adjacent data is recorded as $SKE_{i}$. If the difference of several consecutive angular velocities is greater than 0, it indicates that the upper limb is performing an accelerating movement; on the contrary, if the difference is less than 0, it indicates that the upper limb is performing a decelerating movement. A set of angular velocity data contains *N* sampling points, and all sampling points are rendered different in turn, and *N* − 1 difference values can be obtained. The equation for calculating the difference is shown in Equation (3).

(3) $$SKE_{i}=E_{i+1}-E_{i},i=1,2,3\ldots N-1$$

In a set of data, the magnitude of the difference between the maximum value and the minimum value can represent the magnitude of the fluctuation of a set of data. The larger the difference, the greater the volatility. Therefore, the maximum and minimum values of a set of data are obtained, respectively, and the difference is then recorded as MN. The equation for MN is shown in Equation (4).

(4) $$\mathrm{MN}=\max(E_{i})-\min(E_{i})$$

### 2.4. Data Window Segmentation

**Figure 4:**
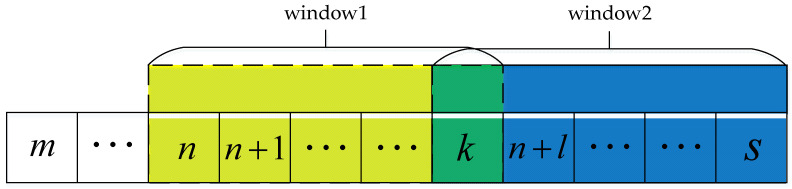
Extraction process of sliding window.

As time accumulates, increasing amounts of inertial data will be collected. If the analysis is carried out with the complete inertial data, the running time of the algorithm will increasingly lengthen. Therefore, this paper uses a sliding window to extract data, and the sliding window extraction process is shown in Figure 4. The length of the sliding window is set to l, and a first-in, first-out strategy is used. In the recognition of action type and motion state, the recognition result of the sliding window is used as the recognition result of the last sampling point in the window. For a sliding window, each time a sampling point is discarded, a new sampling point is added. Considering the delay of controlling the manipulator, combined with the actual process of collecting data, the length of the sliding window is set to 10 in this paper.

## 3. Implementation of Key Models and Methods

The action intention of the upper limb includes what action the upper limb performs and how the upper limb performs the action. The purpose of this paper is to render the manipulator able of completing foot movements as flexibly and accurately as the human hand. Therefore, the research content of this paper is divided into two parts: (1) apply the DTW algorithm based on motor unit to predict the action type of the upper limb; (2) use the GRNN algorithm to identify the grasping action intention of the upper limb to control the manipulator grabbing objects.

### 3.1. DTW Algorithm Based on Motion Units

The foot movements studied in this paper include putting on socks, putting on shoes, and tying shoelaces. Different action types are comprised of different action steps. Therefore, distinguishing the types of foot movements is the key to identifying upper limb movement intentions. There are two difficulties in this research:

(1) It is necessary to predict the type of action during the movement of the upper limb. In the process of controlling the manipulator, the action of the manipulator has high real-time requirements. Therefore, the upper limb action intention studied in this paper is different from the previous action recognition. Action recognition is a “posteriori judgment”, that is, after the action is completed, the action type is identified. However, the intention recognition studied in this paper belongs to a “priori judgment”, that is, in the process of action, the type of action is identified to predict the intention of the upper limb. Solving the problem of action type prediction represents one of the research difficulties.

(2) It is necessary to predict the action intention of the upper limb based on less data sets. As mentioned above, in the movement process of the upper limb, the earlier the action intention of the upper limb is predicted, the better the effect of the recognition method is. The capacity of the dataset greatly affects the performance of the learning algorithm. Therefore, in the case of a small dataset, improving the accuracy of recognizing action intent presents another difficulty in research.

There have been many machine learning algorithms successfully applied in the field of action recognition. The performance of machine learning algorithms largely depends on a large number of datasets, and the computational cost is high. Moreover, most machine learning algorithms identify the type of upper limb movement after the movement is completed. After comparing many action recognition methods, this paper selects the DTW algorithm to predict the action types of the upper limb.

The DTW algorithm is a method employed to calculate the difference between two time series. When the lengths of the two time series are different, the DTW algorithm can also obtain the optimal value by adjusting the relationship between the corresponding elements at different time points of the time series. As a result, it bears a wide range of applications in the field of action recognition. Guoquan Li et al. [26] used the DTW algorithm to identify the motion patterns of smart terminals, which improved the positioning accuracy of the pedestrian dead reckoning system. Xu Leiyang et al. [27] implemented a dynamic time warping algorithm to calculate the minimum distance between two node trajectories, and realized the classification of Taijiquan actions.

The essential function of the DTW algorithm is to calculate a set of actions with the smallest error by comparing the current motion data with the motion data of all standard actions in the template library. The Euclidean distance is usually calculated to measure the similarity between motion data and template data. The basic idea of DTW algorithm is as follows:

Given a reference template represented as

(5) $$R=\left[r_{1},r_{2},\cdots ,r_{m},\cdots ,r_{M}\right]$$

where *M* represents the total number of eigenvalues contained in the reference template, and $r_{m}$ is the mth eigenvalue.

The test template is represented as

(6) $$T=[t_{1},t_{2},\cdots ,t_{n},\cdots ,t_{N}]$$

where *N* represents the total number of eigenvalues contained in the test template, and $t_{n}$ is the *n*-th eigenvalue.

The calculation formula of the distortion degree between the motion data and the template data is:

(7) $$D[T(n),R(m)]={\left(t_{n}-r_{m}\right)}^{2}$$

Finally, the overall distortion *D*[*T*,*R*] is used to judge the similarity between the motion data and the template data. Since the total number of eigenvalues in the reference template is not necessarily equal to the total number of eigenvalues in the test template, there are two cases when calculating the overall distortion degree *D*[*T*,*R*]:

(1) *N* = *M*, the total number of eigenvalues in the reference template is equal to the total number of eigenvalues in the test template. Calculate the distortion of *n* = *m* = 1,⋯, *n* = *m* = *N* in turn and take the sum, that is

(8) $$D[T,R]=\sum_{n=m=1}^{n=m=N}{\left(t_{n}-r_{m}\right)}^{2}$$

(2) *N* ≠ *M*, that is, the total number of eigenvalues of the two is not equal. The smaller patterns are mapped onto the larger pattern sequence by some expansion method. Then, the distortion between the new corresponding eigenvalues is calculated separately, so as to obtain the total distortion *D*[*T*, *R*].

**Figure 5:**
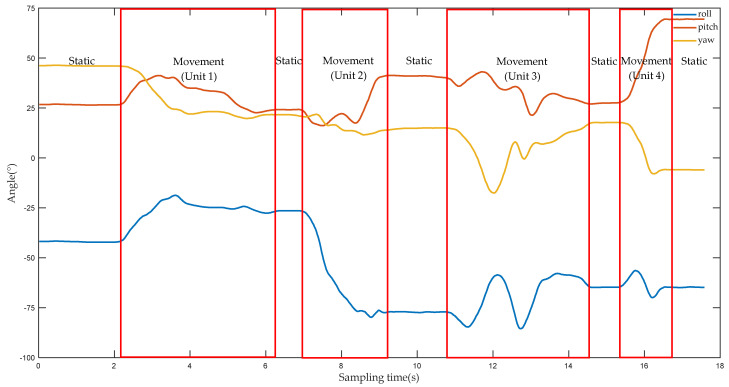
The angle change values of the three axes of the palm.

However, by analyzing the experimental data, at least 15 s was required to finish a complete action. Since the sampling frequency of the device is 50 Hz, at least 800 sampling points will be obtained during the process of collecting data. In the course of using the DTW algorithm, problems arise if the complete sample points are compared to all reference templates. With the accumulation of time, the number of sampling points will gradually increase, and the algorithm will consume more time. However, the control of the manipulator bears the requirement of low latency, and the processing process of large amount of data clearly cannot meet the control requirements of the manipulator. Based on the above analysis, this paper proposes a DTW algorithm based on motion unit. Figure 5 shows the angle change values of the three axes of the hand as the upper limb performs the action of putting on socks. When the amplitude of the angle values of several consecutive sampling points no longer changes, it means that the upper limb is in a static state, such as in the range of the sampling time (0–2 s). When the fluctuation range of the angle values of several consecutive sampling points varies greatly, it indicates that the upper limb are in an movement state, such as within the range of the sampling time (4–6 s). Therefore, the static state of the upper limb is defined as the “static segment”, and the movement state of the upper limb is defined as the “movement segment”. The two states are determined as follows:

(1) In the sliding window, first calculate the difference between all two adjacent sampling points, and calculate the absolute value. Then, compare all the absolute values to identify the one with the largest value;

(2) If for the three parts of the upper limb, the maximum values of the three axial directions are all less than 0.3°, then the motion state of the upper limb is static at this time, and the window is the static segment;

(3) For the three parts of the upper limb, if the maximum value of the three axial directions exceeds 1°, then the motion state of the upper limb at this time is motion, and the window is the movement segment.

**Figure 6:**
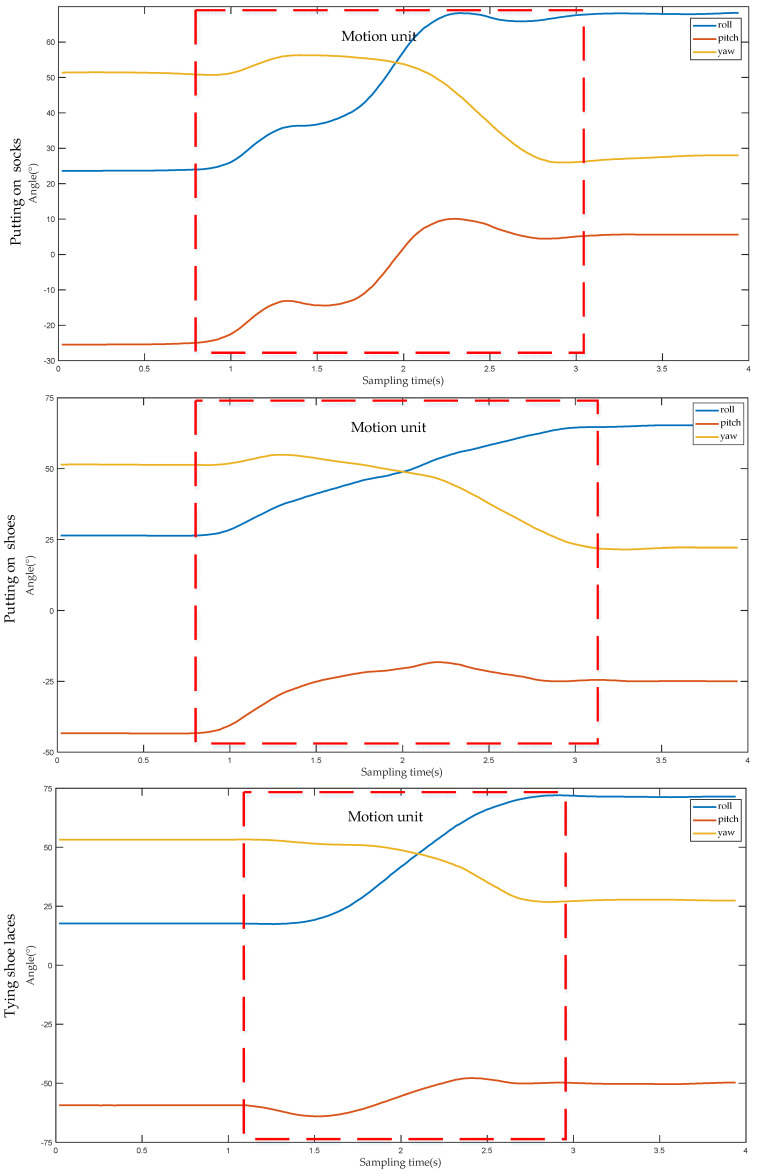
The angle change curve of the first motion unit of the forearm under the three action types.

A complete action can be composed of several static segments and movement segments. For example, the action of putting on socks in Figure 6 includes five static segments and four movement segments. The movement segment of the upper limb is called a motion unit, and then a complete action can be divided into several motion units. The length of a motion unit depends on the duration of the action. In Figure 5, a movement segment includes at least 50 sampling points, which is equivalent to 1 s. The duration of the movement segment is relatively small relative to the duration of the full action. If only movement segments are identified, the time consumed by the algorithm will be greatly reduced. Therefore, when using the DTW to identify the type of action of the upper limb, the object of analysis is the motion unit, not the time series.

Considering the real-time requirement of controlling the manipulator, this study only selects the first motion unit and the second motion unit of a complete action for analysis. Figure 6 illustrates the angle change curve of the first motion unit when the lower performs three different actions. Whether putting on socks, putting on shoes, or tying shoelaces, the action represented by the first motion unit refers to the movement of the upper limb from the initial position to the vicinity of socks, shoes or shoelaces. Among them, the initial positions of the upper limbs are all at the waist; and the socks, shoes and shoelaces are all located at the same position, indicating that the initial and destination positions of the upper limb are the same. During the process from the initial position to the target position, the direction of the angle change of the lower is also the same for the three different actions. Therefore, the angle values of each part of the upper limb before and after the first motion unit are recorded, respectively, and then the difference between the two can be used to obtain the angle change value of each part of the upper limb when this motion unit is performed, as shown in Table 1. It can be observed from Table 1, that for the same part, although the action type is different, the positive and negative angle values in three directions are the same, that is, the angle values in all directions change in the same direction, and the difference in angle values is caused solely by the amplitude of upper limb movement. Combined with the data in Table 1, it is shown that for different action types, all parts of the upper limb move in the same direction when carrying out the first motion unit. Therefore, the type of action of the upper limb cannot be distinguished effectively by the first motion unit alone.

**Figure 7:**
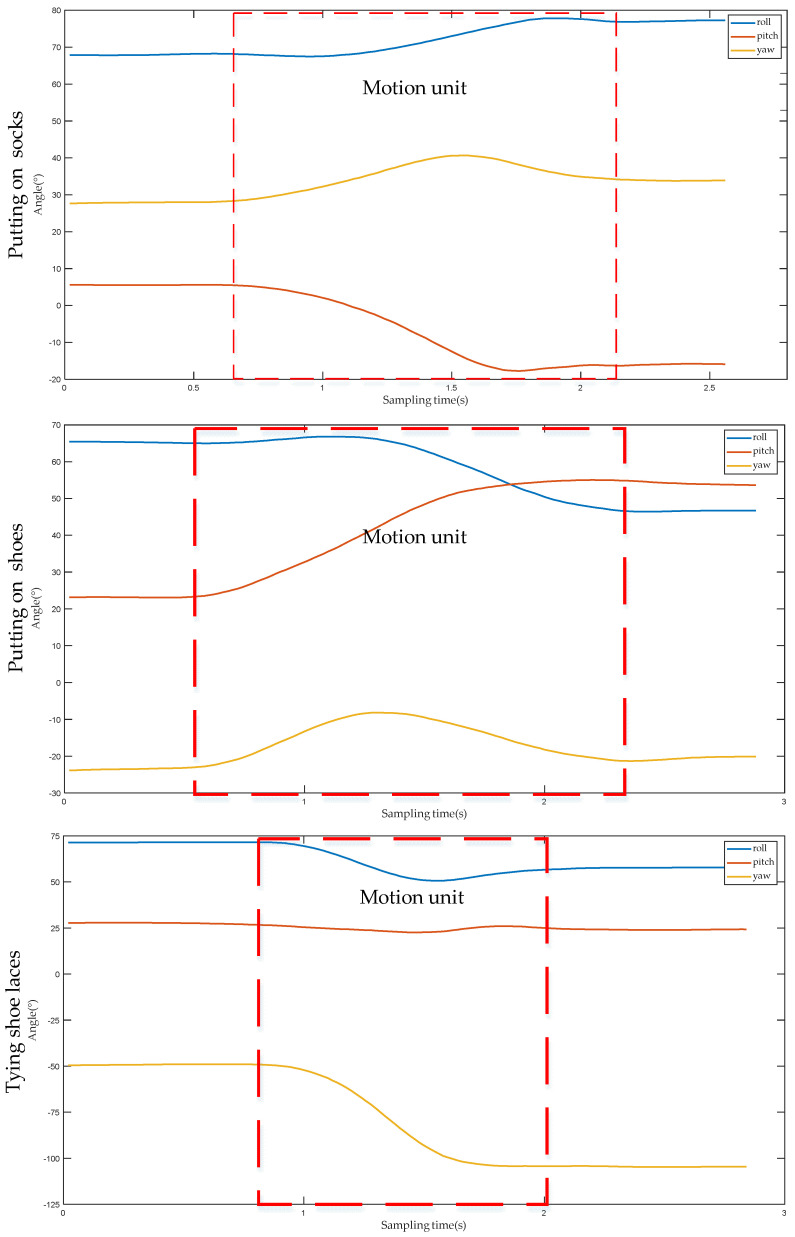
The change curve of the angle of the second motion unit of the forearm under the three action types.

Figure 7 shows the angle change curve of the second motion unit when the lower arm performs three different actions. Although the initial positions of putting on socks, putting on shoes and tying shoelaces are the same, due to the different target positions of each action, the angle changes of each part of the upper limb are also different. For example, for the hand, the angle value of the yaw angle changes greatly when putting on socks, the angle value of the pitch angle changes greatly when putting on shoes, and the angle value of the roll angle changes greatly when tying shoelaces. Table 2 displays the angle change values of each part of the upper limb after performing the second motion unit under three different action types. It can be seen from Table 2 that for different actions, the change range and direction of the angle value of each part of the upper limb are different. Therefore, according to the first motion unit and the second motion unit, the DTW algorithm can be used to distinguish the action types of the upper limb.

**Figure 8:**
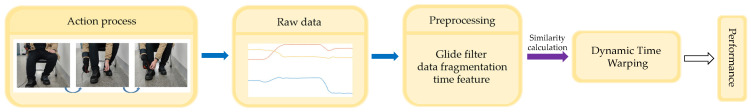
Recognition model of DTW algorithm based on motion unit.

The recognition model of the DTW algorithm based on the motion unit is shown in Figure 8. First, the movement data of each part of the upper limb is collected, and the angle data is processed by moving average filtering and data segmentation. Then, the angle change values before and after the first motion unit and the angle change values before and after the second motion unit are recorded to construct a feature vector. Secondly, the DTW algorithm is used to calculate the similarity between the feature vector and the standard vector of the template library, and the smallest similarity value represents the action type of the current upper limb. For three different action types, 350 datasets under each action type are taken, and the angle change values from the first motion unit to the second motion unit are calculated separately. For the angle change value of the same motion unit, 350 data are summed first, and then the average value is calculated. The obtained data is the standard data of each action type, and the standard vector of the template library is shown in Table 3.

### 3.2. GRNN Algorithm Based on 10-Fold Cross Validation

Predicting the action types of the upper limb is mainly used for manipulator control. The key to controlling the manipulator is to accurately judge the user’s grasping intention, and to control the manipulator’s fingers to open or close in time. Generally speaking, the grasping action of the human hand generally occurs after the upper limb is static. Grasping and releasing objects are essentially controlling finger movements, and the control methods are the same. Thus, the action rules of grasping objects are also applicable to the actions of releasing objects. Based on these understandings, we analyze the movement rules of human grasping objects based on the changes in the movement state of the upper limb. Whether putting on socks, putting on shoes, or tying shoelaces, the essence of these actions is grabbing or releasing objects. A complete action can be divided into several grasping actions. Analyzing the action law of a single grasping action is helpful to design the control method of the manipulator.

**Figure 9:**
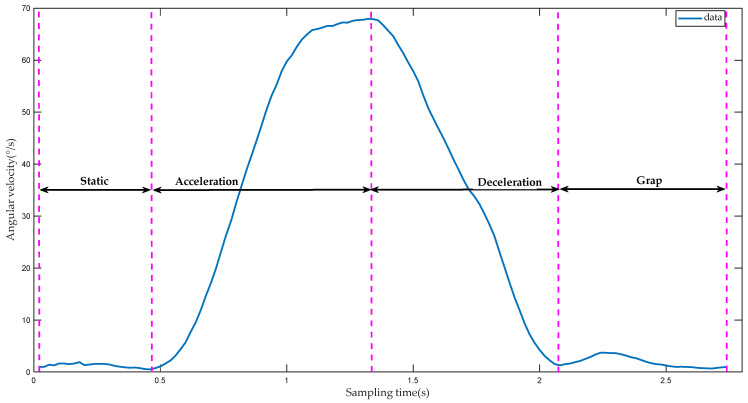
The comprehensive value of the angular velocity of the hand during the grasping process.

Figure 9 shows the change curve of the comprehensive value of the angular velocity of the hand during the grasping process of the human hand. In Figure 9, the first segment is defined as a “static segment”. This segment is the stage in which the upper limb is in a static state before the grasping action. At this time, the comprehensive value of the angular velocity of the hand bears only a small fluctuation around a 0 value. The second and third sections are defined as the “acceleration section” and “deceleration section”. Both the acceleration segment and the deceleration segment represent the stage when the upper limb moves toward the object. The comprehensive value of the angular velocity of the hand first increases and then decreases, with a clear process of first accelerating and then decelerating. The fourth segment is defined as the “grab segment”. This segment represents the phase in which the fingers are closed to grasp the object when the upper limb is static. Due to the movement of the fingers, the integrated value of the angular velocity of the hand fluctuates, but when the fingers are closed, the integrated value of the angular velocity of the hand remains only around 0.

In the above analysis of the comprehensive value of the hand angular velocity during finger grasping, it can be observed that the motion state of the upper limb includes static, acceleration and deceleration, and the grasping action generally occurs in the static state following the deceleration state. According to the fundamental principles of kinematics, it can be concluded that the acceleration state of an object appears only after the static state, and the deceleration state only appears following the acceleration state. Therefore, the control method of the manipulator is designed as follows: the motion process of the upper limb is divided into static, acceleration, and deceleration. In a set of motion data, when the upper limb is detected as reaching a static state following the deceleration state, the manipulator is programmed to open or close. It needs to be clarified that at this time, the upper limb has already experienced the acceleration state by default.

This study employs a GRNN to identify the motion states of the upper limb. GRNN is a general non-parametric regression model that approximates the objective function by activating neurons, and bears stronger advantages than RBF in local approximation, global optimization and classification performance [28]. The GRNN converges on the optimized regression surface with a large number of data samples. When the number of samples is small or the sample data is unstable, the regression prediction results are extremely clear. In a GRNN model, the smoothing parameter is the only parameter that can be implemented to optimize the network model. The selection of smoothing parameters includes two methods, one is to use manual adjustment, which is simple and easy to operate, but exhibits low efficiency and poor accuracy; the other is to use an algorithm to obtain the optimal value [29]. In this study, the 10-fold cross-validation method was used to select the optimal smoothing parameters of the GRNN according to the minimum mean square error.

**Figure 10:**
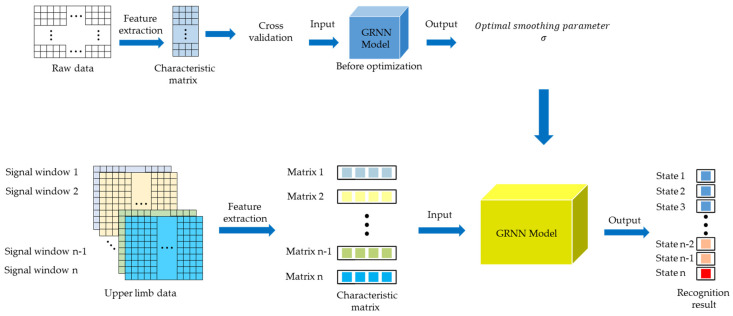
GRNN algorithm model based on 10-fold cross-validation.

In summary, this paper proposes a GRNN algorithm model based on a 10-fold cross-validation to identify the motion states of the upper limb, as shown in Figure 10. The algorithm is divided into the following two processes: (1) The experimental data is divided into training set and test set, and the optimal smoothing parameters of GRNN are selected by 10-fold cross-validation method. (2) According to the collected angular velocity data of each part of the upper limb, the optimized GRNN is used to identify the motion state of the upper limb.

**Figure 11:**
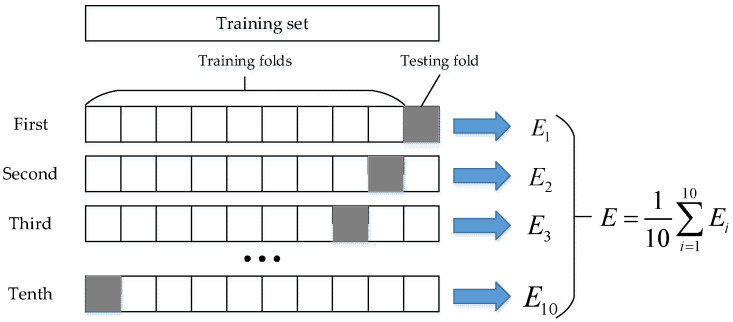
The flow of the 10-fold cross-validation method.

Consider the action of putting on socks as an example. First, use the inertial sensor to obtain the raw data, including the angular velocity data of the x-axis, y-axis, and z-axis of the upper arm, lower arm and hand, and record the motion state of the upper limb. Among them, “1” represents the acceleration state of the upper limb, “−1” represents the deceleration state of the upper limb, and “0” represents the static state of the upper limb. After filtering, the method of time domain feature extraction is employed to extract characteristic parameters such as variance, difference, maximum value, and minimum value, to construct a feature vector. The matrix vector contains 4 columns. The motion state of the upper limb was used as the state vector, and the state vector and feature vector were randomly divided into 10 parts, 9 of which were used as training data, and the other part was used as test data. The average accuracy of the results of 10 iterations of training and verification was used as the estimation of the algorithm accuracy. The process of 10-fold cross-validation is shown in Figure 11. Then, the neural network is trained by the 10-fold cross-validation method, and the mean square error is selected to measure the performance of the neural network, so that the optimal smoothing parameter σ can be obtained. According to the training results, the smoothing parameter of the GRNN was set to 0.7. In the process of the manipulator reproducing the action of the human hand, since the length of the signal window is 10, it is necessary to identify the motion state every instance 10 sampling points are obtained. By filtering the data in the sliding window and extracting the feature parameters, the feature matrix vector matrix can be obtained. Input the feature matrix vector into the trained GRNN model, and the output of the model represents the motion state of the current sampling point.

## 4. Experiment and Result Analysis

### 4.1. Experimental Equipment

**Figure 12:**
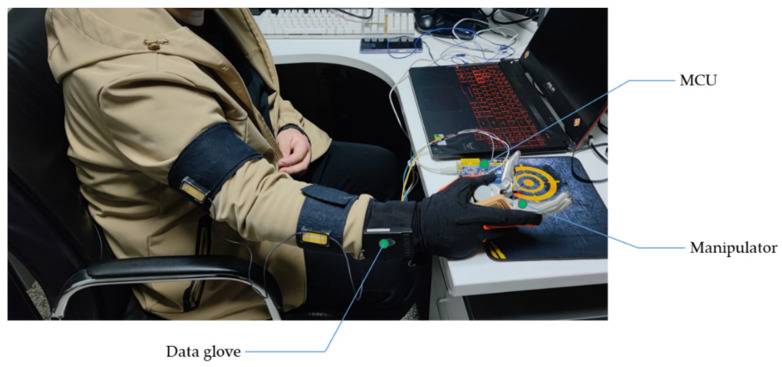
Experimental equipment.

The experimental equipment consists of two parts, as shown in Figure 12. One is the data glove, which is used to collect Euler angles and angular velocities of the upper arm, lower arm and hand. The other is a prosthetic hand, which is connected to a single-chip microcomputer to grab objects. The whole process of data processing and analysis is performed on a computer. When the generalized regression neural network recognizes the static state of the upper limbs, it sends instructions to the microcontroller. At the same time, the single-chip microcomputer controls the disabled hand to open or close the fingers in a high and low level manner.

The classification method was implemented via the MATLAB toolbox. The neural network model was implemented through MATLAB functions. Using the Visual Studio 2019 platform, we developed the host computer software for data acquisition and analysis. Training and testing are performed on a PC with an Intel Core i7-6500U CPU, 12 Gb DDR-III 2400 MHz RAM, and NVIDIA GeForce 940MX.

### 4.2. Activity Definition

**Figure 13:**
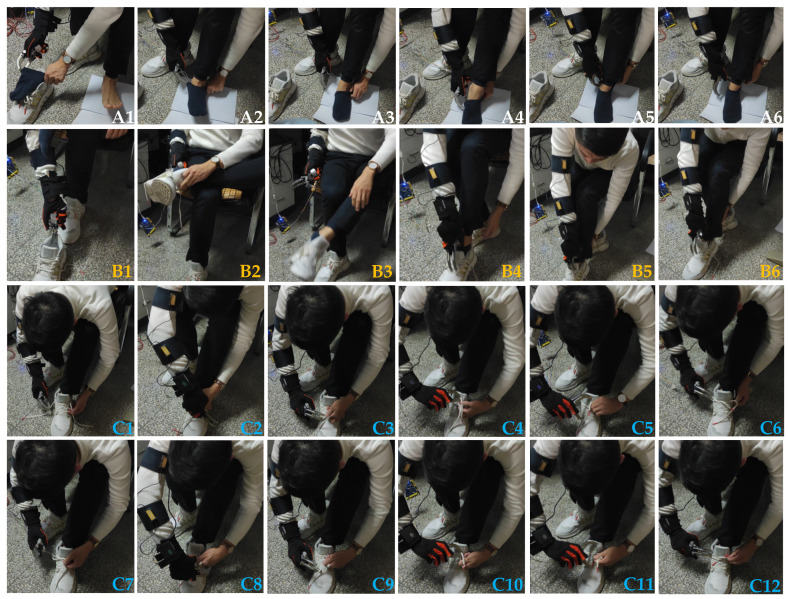
Step composition of three types of actions.

The purpose of the experiment is to verify the feasibility and applicability of the upper limb action intention recognition method by using a manipulator to reproduce human hand actions. The research object of this paper cover the actions of putting on socks, putting on shoes, and tying shoelaces in the legs in daily life, and there are many different steps involved to complete an action. Based on living habits, for three different actions, this paper selects a frequently used step accordingly, as shown in Figure 13, where the first row is the flow chart of putting on socks, the second row is the flow chart of putting on shoes, lines 3 and 4 represent flowcharts of tying shoelaces. The experiment of employing a manipulator to reproduce human hand movements was performed by all volunteers who had previously participated in the experiment. In the experiment of repetitive motion of the manipulator, each volunteer performed the same action as before, and each volunteer performed 20 repetitions of the action. Similarly, the movement data of each part of the upper limb was stored when the manipulator reproduced the action. It should be noted that the steps involved for the human hand to complete the leg action are the same as the steps for the prosthetic hand to complete the leg action, and both follow the steps as observed in Figure 13.

### 4.3. Experimental Results of Dynamic Time Warping Algorithm Based on Motion Units

**Figure 14:**
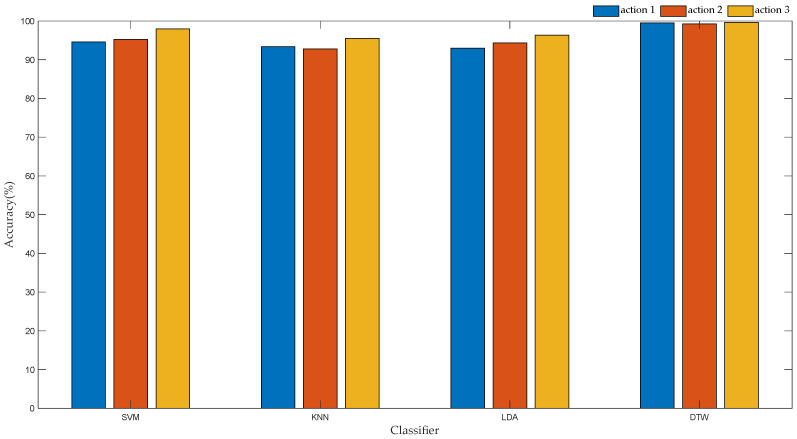
Accuracy rate of each classifier recognition.

To verify the recognition effect of the DTW algorithm based on motion unit, we used support vector machine (SVM), k-nearest neighbor algorithm (KNN), linear discriminant analysis (LDA) and DTW for data modeling. First, for different action types, the angle change values of each part before and after the first motion unit are recorded to construct feature vectors. The actions are denoted as follows; putting on socks as “1”, the action of putting on shoes as “2”, and the action of tying shoelaces as “3”, to construct the state vector. Then, the feature vector and state vector are divided into 10 parts, of which 7 are used as training set and 3 are used as test set. The training set is used to train the model, and the test set is employed to test the classification effect of the model. The recognition accuracy of each classifier is shown in Figure 14. The time complexity of each classifier is given in Table 4.

**Figure 15:**
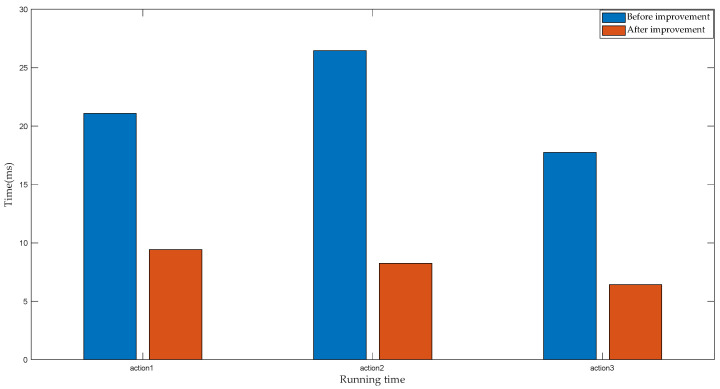
Running time of the two algorithms.

In order to compare the effect of the improved DTW algorithm on the running time, the traditional DTW algorithm and the optimized DTW algorithm are compared. For three different action types, 10 experimental samples were obtained for testing, and the running time of each algorithm recognition was recorded. MATLAB software was used to calculate the running time of the algorithm. Figure 15 and Table 5 show the average running time for both algorithms to recognize the same action.

By analyzing the data in Figure 15 and Table 5, the following experimental conclusions can be drawn:

(1) For the same action type, the DTW algorithms based on motion units exhibit better classification performance, with an average recognition accuracy of 99.46%.

(2) For the same classifier, the classification effect of Action 3 bears a higher recognition rate than Action 1 and Action 2. This is because the second motion unit of Action 3 has a short duration, so that the angle of change of each part is smaller, thus becoming easier to distinguish. The identification results are in line with the actual experimental situation.

(3) Compared with the DTW algorithm before optimization, the running time consumed by the optimized DTW algorithm was shorter, and the average running time measured 8.027 ms, representing 2/5 of the sampling time. However, the average running time of the DTW algorithm before optimization measured 21.762 ms, thus exceeding the sampling time.

Therefore, regarding both the recognition accuracy and the running time of the algorithm, the time warping algorithm based on the motion unit exhibits better performance.

### 4.4. Analysis of Recognition Results of GRNN Based on 10-Fold Cross Validation

**Figure 16:**
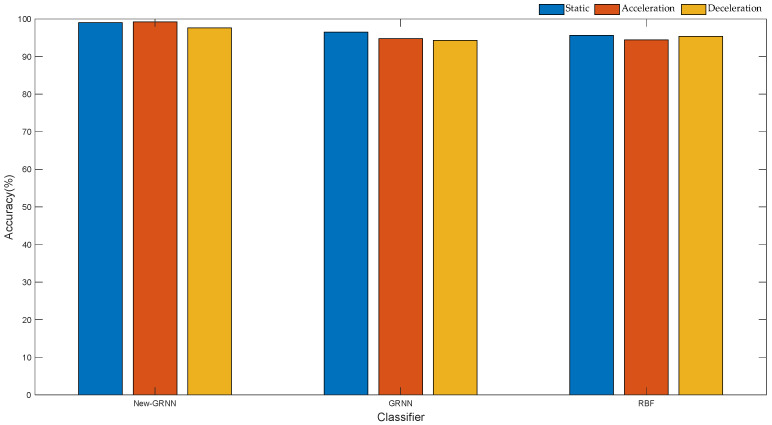
Classification effects of three neural networks.

To measure the recognition effect of the GRNN based on 10-fold cross-validation, based on the same training set and test set, we used the traditional GRNN, RBF and the optimized GRNN to build a classifier. For three action types, 100 datasets were randomly selected, 70 of which were used as training set and 30 as test set. Feature extraction is first performed on the raw data to construct feature vectors. The corresponding motion state is selected as the state vector. The feature vector and state of training are set to train the three neural networks, respectively, and finally the trained neural network model is applied to classify the data of the test set. The classification results are shown in Figure 16 and Table 6.

By analyzing the data in Figure 16 and Table 6, the following experimental conclusions can be drawn:

(1) Among the three classifiers, the GRNN based on 10-fold cross-validation exhibits higher recognition accuracy, with an average recognition rate of 98.28%.

(2) For the same data set, there are certain differences in the classification performance of the three neural networks. The order from largest to smallest; optimized GRNN > traditional GRNN > RBF. This also shows that the GRNN displays better classification performance than RBF.

(3) Compared with the classification results of action types, the results of neural network recognition of motion states are lower than the former. This is because for the same motion state, the data changes shown by the upper arm, the lower arm, and the hand are different. For example, from the deceleration state to the static state of the upper limb, the angular velocity data of the upper arm and the lower arm reach closer to 0 faster than the angular velocity data of the hand.

## 5. Discussion

This paper incorporates the daily activities of healthy people as the research object. The motion data of the upper limb were collected by inertial sensors, and the action intentions of the upper limb were analyzed and recognized. First of all, based on the behavioral habits of people’s daily actions, this paper presents a movement procedure for putting on socks, putting on shoes and tying shoelaces. In order to assist handicapped individuals to complete basic daily life activities, this paper proposes a DTW algorithm based on motion units. The angle data of the upper limb are preprocessed, including filtering, sliding window segmentation and feature extraction. Then, based on whether the upper limb is moving, the complete motion data are divided into several motion units. The angle change values of each part in the first and second motion unit are calculated to construct the feature vector of the input classifier. Second, the classification results are compared on multiple learned classifiers using the same input vector. Experimental data show that, compared with SVM, LDA and KNN, the DTW based on motion unit exhibited better classification performance, and the accuracy of action recognition measured the highest, reaching 99.46%. Moreover, in terms of the running time of the algorithm, the traditional and improved DTW algorithms were compared. The average running time of the DTW algorithm based on motion unit measured 8.027 ms, representing 2/5 of the sampling time, and 1/3 of the running time of the DTW. In order to enable the manipulator to reproduce the human hand movements of healthy people, a GRNN algorithm based on 10-fold cross-validation was designed in this paper to control the manipulator to grasp objects. The motion state of the upper limbs was divided into acceleration, deceleration and static, and the static state following the deceleration state was employed as the symbol for controlling the manipulator. First, based on the angular velocity data of the upper limb of healthy people, a 10-fold cross-validation method was implemented to select the optimal smoothing parameters of the neural network according to the minimum mean square error. Then, when preprocessing the angular velocity data of the upper limb, the input vector of the neural network was constructed by extracting characteristic parameters such as variance, maximum value, minimum value, and difference, and the recognition performance of different neural networks was compared. The experimental results reveal that, compared with the traditional GRNN and RBF, the optimized GRNN shows better performance, and the recognition accuracy rate reaches 98.28%. Finally, the actions of putting on socks, putting on shoes and tying shoelaces were reproduced using the manipulator, resulting in good demonstration results.

Compared with the use of EMG signals to control the prosthetic hand, the method employing the motion information of the upper limb bears certain advantages. Whether it be a normal person’s upper limb or a prosthetic limb, the inertial sensor collects the motion information of the limb during the movement process, regardless of the shape of the limb. In addition, this method can remove the influence of the degree of muscle contraction. For subjects of different ages or genders, there may be some differences in the degree of muscle contraction. However, this difference did not affect the motor information of the upper limbs.

Although the method designed in this paper can control the manipulator to reproduce the action of the human hand well, there still remain some problems that require further improvement and in-depth research. The human hand movements studied in this paper were limited to the opening or closing of fingers, and in people’s daily movements, some movements require more degrees of freedom to complete. When designing the control method of the manipulator, the motion state of the upper limb is determined by the threshold analysis method, and there is a certain time difference concerning the real motion state of the upper limb, thus there exists at least a 1.5 s delay in the process of controlling the manipulator. In the experimental section, the method of a healthy participant holding a prosthetic hand was adopted, in which it was assumed the subjects had lost the palmar surface of the hand, and the prosthetic hand was employed as a substitute. Although this method verifies the feasibility of the algorithm, there remains a long pathway before it can be extended to disability-related applications. Therefore, future research will focus on the high dexterity and low latency of manipulator control and apply the proposed approach to services for individuals with disabilities.

## Figures and Tables

**Table 1:** The angle change value of each part when the first motion unit is performed.

Body Parts	Putting on Socks			Putting on Shoes			Tying Shoe Laces		
	Δroll	Δpitch	Δyaw	Δroll	Δpitch	Δyaw	Δroll	Δpitch	Δyaw
**Upper arm**	0.253	−0.761	0.779	0.117	−0.488	0.342	0.339	−0.654	0.170
**Lower arm**	0.887	0.622	−0.481	0.777	0.380	−0.563	1.122	0.237	−0.551
**Hand**	0.196	0.511	−1.273	0.078	0.552	−1.077	0.181	0.739	−1.219

**Table 2:** The angle change value of each part when the second motion unit is performed.

Body Parts	Putting on Socks			Putting on Shoes			Tying Shoe Laces		
	Δroll	Δpitch	Δyaw	Δroll	Δpitch	Δyaw	Δroll	Δpitch	Δyaw
**Upper arm**	0.232	−0.050	−0.504	−0.079	0.561	0.261	−0.445	−0.051	−1.429
**Lower arm**	0.177	−0.436	0.125	−0.396	0.053	0.613	−0.059	−1.092	−0.278
**Hand**	0.039	0.141	−0.204	−0.334	0.238	0.494	−0.291	−0.135	−0.714

**Table 3:** Standard data of template library.

Body Parts		Wearing Socks		Wearing Shoes		Tying Shoe Laces	
		Unit 1	Unit 2	Unit 1	Unit 2	Unit 1	Unit 2
**Palm**	Δroll	0.01	0.20	0.10	0.10	0.20	−0.55
	Δpitch	−0.50	−0.15	−0.60	0.55	−0.73	−0.05
	Δyaw	−0.30	−0.52	−0.10	0.30	−0.10	−1.70
**Forearm**	Δroll	0.60	0.25	0.35	−0.30	0.90	−0.30
	Δpitch	−0.55	0.10	−0.8	0.60	−0.55	−0.10
	Δyaw	0.40	−0.30	0.45	0.10	0.15	−1.20
**Shoulder**	Δroll	0.15	0.12	0.00	−0.35	0.20	−0.20
	Δpitch	0.55	0.10	0.70	0.30	0.60	0.00
	Δyaw	−1.3	−0.15	−1.2	0.37	−1.30	−0.50

**Table 4:** Time complexity of each classifier.

	SVM	KNN	LDA	DTW
**Computational complexity**	$O\left(n^{2}\right)$	O(n)	$O\left(n^{2}\right)$	$O\left(n^{2}\right)$

**Table 5:** Comparison of the running time of the two algorithms.

Classification	Action 1	Action 2	Action 3
	Running Time (ms)	Running Time (ms)	Running Time (ms)
Before improvement	21.091	26.461	17.733
After improvement	9.422	8.242	6.418

**Table 6:** The accuracy of three neural network classification.

Classification	Static	Acceleration	Deceleration
	Accuracy	Accuracy	Accuracy
**New GRNN**	99.03%	99.23%	97.66%
**GRNN**	96.54%	94.76%	94.33%
**RBF**	95.67%	94.48%	95.37%

## Data Availability

The data presented in this study are available on request from the corresponding author.
